# *EGFR* mutations and high PD-L1 expression of lung squamous cell carcinoma patients achieving pCR following neoadjuvant immuno-chemotherapy: Case report

**DOI:** 10.3389/fonc.2022.1008932

**Published:** 2022-10-19

**Authors:** Xiangyu Xu, Zixia Shi, Dan Fu, Depei Huang, Zheng Ma

**Affiliations:** ^1^ Department of Thoracic Surgery, Chongqing General Hospital, Chongqing, China; ^2^ The Medical Department, 3D Medicines Inc., Shanghai, China

**Keywords:** neoadjuvant immunotherapy, lung squamous cell carcinoma, epidermal growth factor receptor (EGFR), mutations, pembrolizumab, programmed death ligand-1 (PD-L1)

## Abstract

The treatment of lung cancer has fully entered the era of immunotherapy, which has significantly elevated the survival rate of patients with advanced non-small cell lung cancer (NSCLC), thus shedding light on resectable NSCLC. Previous clinical trial data suggested that neoadjuvant immuno-chemotherapy obtained a significant objective response rate (ORR) and disease control rate (DCR). Here, a case that achieved an excellent outcome following neoadjuvant immuno-chemotherapy was reported. The patient admitted to our hospital was 58 years old, female, with a rare case of stage IB lung squamous cell carcinoma (LUSC) harboring both epidermal growth factor receptor (*EGFR*) p.L858R mutations and high expression of programmed death ligand-1 (PD-L1) (tumor proportion score (TPS)=80%). Her tumor substantially shrunk following two cycles of neoadjuvant immuno-chemotherapy. The patient successively received single-port right upper thoracoscopic lobectomy + mediastinal lymph node dissection, which attained pathologic complete response (pCR). Additionally, the patient had grade 2 myelosuppression during the two cycles, which was treated with polyethylene glycol recombinant human granulocyte colony-stimulating factor (rhG-CSF). The patient was discharged uneventfully without any procedure-related complications. Two courses of adjuvant immuno-chemotherapy were administered postoperatively, leaving the patient in good physical condition at the 5-month follow-up visit. This case provided evidence for the feasibility and effectiveness of neoadjuvant immuno-chemotherapy in treating early-stage LUSC with *EGFR* mutations and high expression of PD-L1. However, randomized and multi-center controlled trials are required to validate the findings.

## Introduction

As the molecular biology of lung cancer advances, immunotherapy and targeted therapy have become standard treatment protocols for advanced lung cancer in clinical practices with their application in early-stage lung cancer explored as well. Several clinical trials have proved the safety and efficacy of programmed death-1 (PD-1)/programmed death ligand-1 (PD-L1) inhibitors of neoadjuvant therapy in resectable non-small cell lung cancer (NSCLC) (1). The Checkmate 816 trial (2) was the first phase III randomized clinical trial to substantiate that neoadjuvant immuno-chemotherapy can significantly raise the percentage of pathologic complete response (pCR) in patients with resectable NSCLC, which was also the first and only preoperative neoadjuvant therapy protocol for lung cancer approved by the Food and Drug Administration (FDA). Moreover, identical to NEOSTAR (3) and NADIM (4) studies, patients with epidermal growth factor receptor (*EGFR*)+/anaplastic lymphoma kinase (*ALK*)+ were excluded and protein expression of PD-LI in baseline tumors was assessed in the Checkmate 816 trial, which revealed the association between up-regulated expression of *PD-L1* and patient’s response to neoadjuvant immunotherapy. The targeted therapy exhibited prominent efficacy in treating NSCLC with *EGFR* mutations, especially the third generation of EGFR-tyrosine kinase inhibitors (EGFR-TKIs), which played essential roles in first/later line treatment and postoperative adjuvant therapy. However, only a few studies concerning neoadjuvant targeted therapy in lung cancer are available. NeoADAURA (5) and CTONG1103 (6) trials confirmed the efficacy and safety of neoadjuvant targeted therapy in patients with *EGFR* mutations, presenting superior clinical outcomes than neoadjuvant chemotherapy, while the effectiveness and safety of *EGFR*-TKIs of the neoadjuvant therapy for NSCLC remain undetermined. Hence, the optimal neoadjuvant therapy protocol is still undefined for NSCLC patients harboring both *EGFR*-sensitive mutations and *PD-L1* positive. Here, we reported a case of stage IB LUSC with *EGFR* mutation, *PD-L1* TPS=80%, and poor preoperative immune microenvironment who achieved pCR following neoadjuvant immuno-chemotherapy.

## Case description

**Figure 1:**
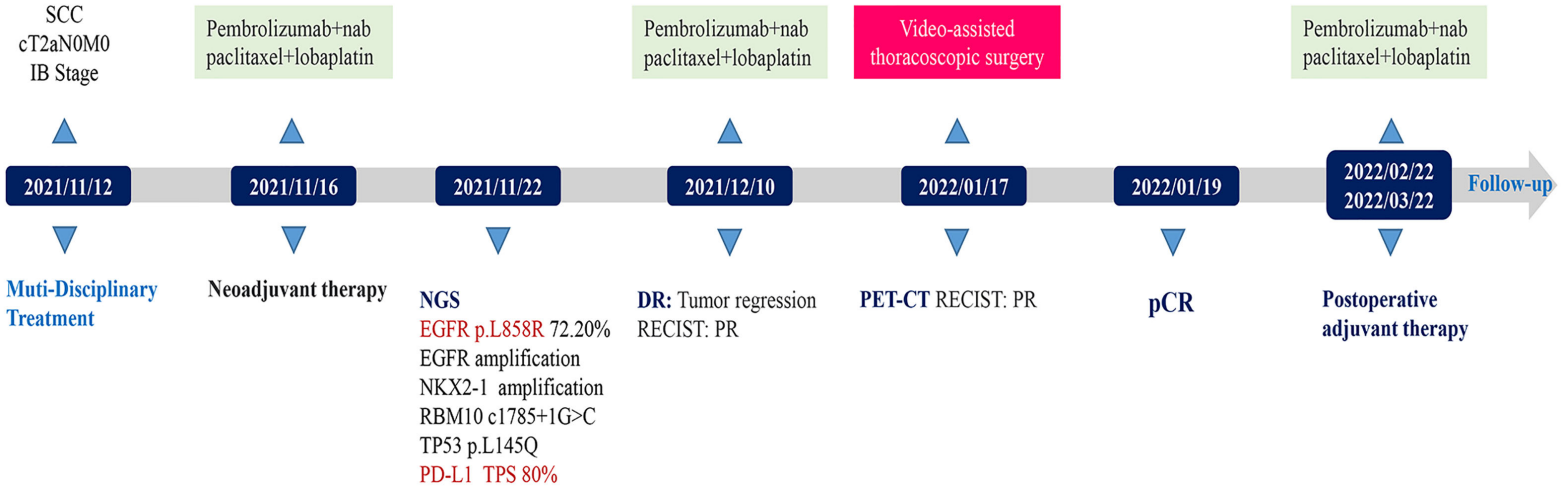
Treatment timeline of the patient.

**Figure 2:**
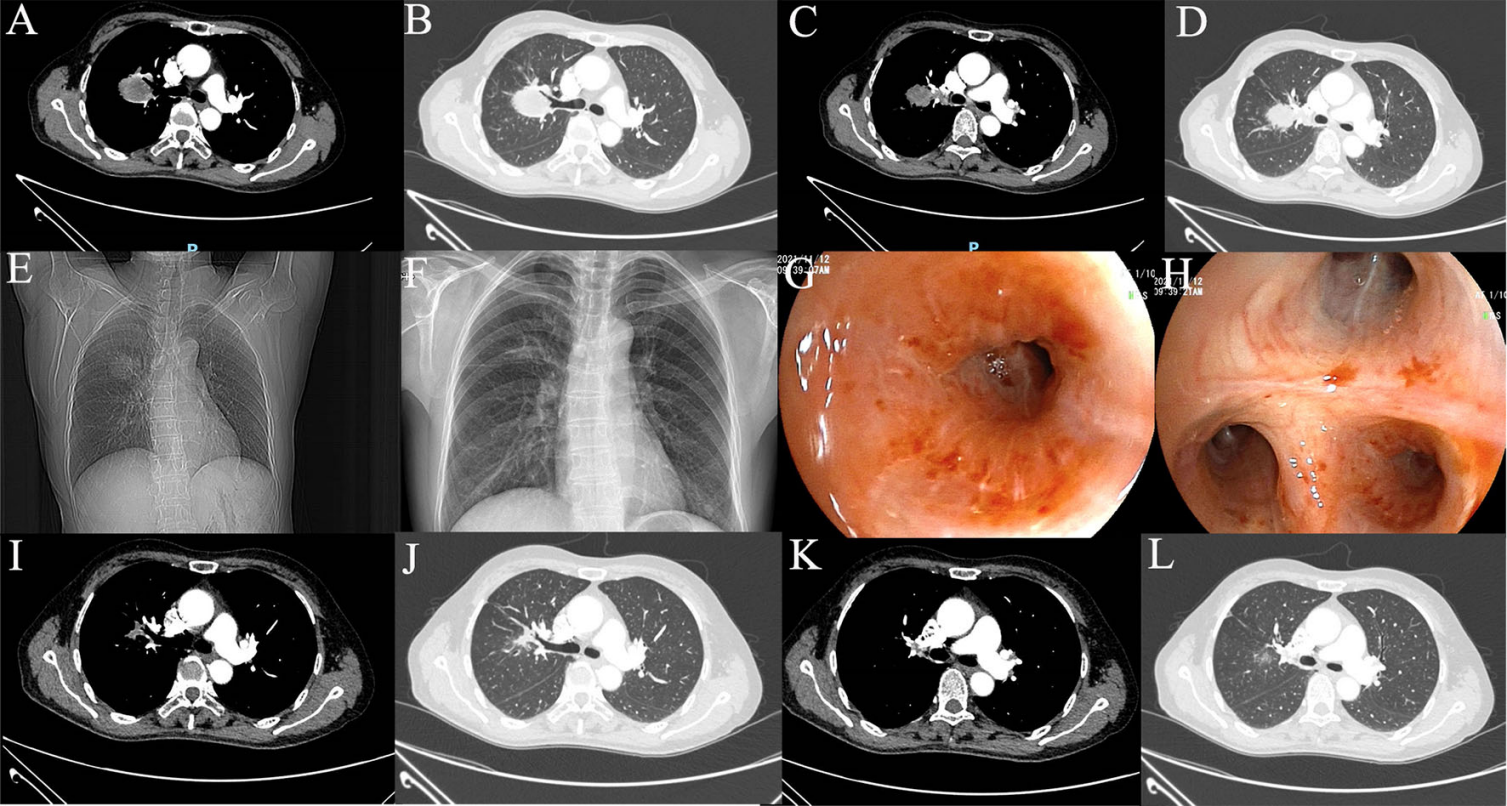
Imaging examination. **(A–D)** Preoperative CT scan; **(E)** Preoperative DR image; **(F)** DR image after one cycle of neoadjuvant therapy; **(G, H)** Preoperative fiberoptic bronchoscopy image; **(I–L)** CT image after 2 cycles of neoadjuvant therapy.

**Figure 3:**
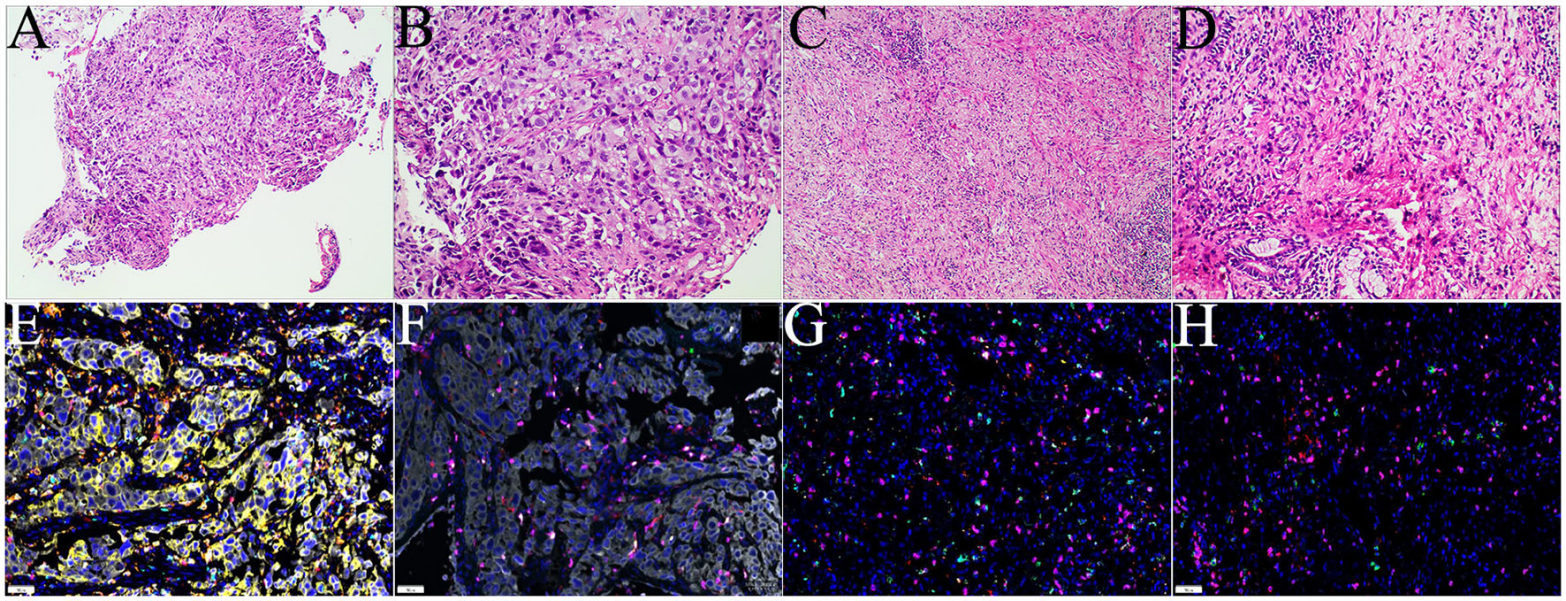
Examinations of pathology and mIHC. **(A, B)**. Histology of biopsies samples (CT guided biopsy);**(C, D)** histology of surgical tissue sample; **(E, F)** mIHC staining of tumor microenvironment of puncture tissue; **(G, H)** mIHC staining of tumor microenvironment after operation.

The 58-year-old patient was female and visited our hospital for one-month hemoptysis with no history of chronic diseases, smoking, and alcohol use (**Figure 1**). The X-ray and enhanced computed tomography (CT) demonstrated a mass in the upper lobe of the right lung (about 3.7 cm × 3.4 cm × 3.5 cm) with lobulated border and burrs, partially-occluded bronchial branches, and there were no obvious enlargement of mediastinal and bilateral hilar lymph nodes, and partial calcification. Central lung cancer was considered (**Figures 2A–D**). Carcinoembryonic antigen (CEA) was 86.98 ng/ml and the cytokeratin 19 fragment (CY21-1) was 4.06 ng/ml. Fiberoptic bronchoscopy indicated neoplasms in the posterior segment of the right upper lung, endoscopic biopsy suggested NSCLC (**Figures 2G, H**), and immunohistochemistry showed P40(+) and TTF-1 (–), suggestive of squamous cell carcinoma (**Figures 3A, B**). A few dyskaryotic cells were detected in bronchoalveolar lavage fluid (BALF). No significant metastasis was identified *via* upper abdominal enhanced CT scan, contrast-enhanced magnetic resonance imaging (MRI) of the head, and whole-body bone emission CT (ECT) scan. Taken together, the patient was classified as cT2aN0M0, stage IB. The patient’s tumor was closely related to the right upper pulmonary artery branch (**Figure 2C**), and the possibility of angioplasty during direct surgery was high, increasing the risk of surgery. There is also the possibility of conversion to thoracotomy, which would be more traumatic for the patient. Considering that the patient was a squamous carcinoma with a low possibility of having driver mutations, the multidisciplinary consultation recommended that neoadjuvant immune combination chemotherapy treatment should be performed first followed by surgery while waiting for NGS test and immunohistochemistry results. After signing the informed consent, the patient received anti-PD-1 therapy plus platinum-based neoadjuvant therapy (intravenous injection of 200 mg Pembrolizumab, 30 mg/m^2^ lobaplatin + 260 mg/m^2^ nab-paclitaxel, D1, q3w). The patient developed grade 2 myelosuppression during therapy, which was treated with an injection of polyethylene glycol recombinant human granulocyte colony-stimulating factor (rhG-CSF) with no grade 3-5 adverse events (AEs) observed. In the meantime, next-generation sequencing (NGS) and immunohistochemical(IHC) detected *EGFR* p.L858R mutations (abundance 72.2%), PD-L1 expression (TPS=80%, CPS(Combined Positive Score)=80), and tumor mutational burden (TMB, 5.03Mut/Mb), accompanied by EGFR copy-number amplification as well as multiple gene mutations, such as *NKX2-1* and *TP53*. Considering the occurrence of *EGFR* mutation, the followed therapy was dilemma, which dependent on the outcome of the first treatment session as informed before. After one course of neoadjuvant immunochemotherpy, CEA and CY21-1 were 23.17 ng/ml and 2.43 ng/ml, respectively, significantly lower than the pre-treatment levels. In the meantime, chest digital radiography (DR) showed a reduction of the right upper lung occupancy (**Figures 2E, F**), implying partial radiographical response. So, the second cycle immunochemotherapy was perfomed as plan. Three weeks later, subsequent to the application of the second course of neoadjuvant therapy, a chest X-ray and enhanced CT scan were performed, indicating a marked reduction of the lesion in the right upper lung(about 1.9 cm × 1.4 cm) with partially bronchial stricture. At this point, CEA and CY21-1 were 3.06 ng/ml and 1.81 ng/ml respectively. Besides, positron emission tomography-computed tomography (PET-CT) illustrated an irregular patchy hyperdense shadow (2.76 cm × 2.18 cm × 1.59 cm) near the hilum of the right lung upper lobe and a mild increase in radioactivity uptake (maximum SUV = 2.57, mean SUV = 2.37), indicating that the tumor activity was remarkably suppressed (**Figures 2I-L**). Partial response (PR) was assessed according to the Response Evaluation Criteria in Solid Tumors (RECIST) version 1.1. In the fifth week after the neoadjuvant therapy, the single-port right upper thoracoscopic lobectomy + mediastinal lymph node dissection was successfully performed, during which the blood loss was approximately 50 ml. Postoperative pathology (**Figures 3C, D**)showed that the mass was 2.5 cm × 2 cm × 1.5 cm and that the fibrous tissue of the tumor bed exhibited hyperplasia with degeneration. Lymphocytes infiltrated the areas containing multinucleated giant cells and foam cells without residual disease; the bronchial mucosa manifested chronic inflammation without residualdisease. Efficacy assessment of neoadjuvant therapy showed that pCR was achieved. The lymph nodes of 2R (0/2), 3A (0/2), 4R (0/1), group 7 (0/1), group 10 (0/1), group 11 (0/7), and group 12 (0/1) were disease-free. The bronchial stump of the right upper lung was also free of cancer cells. The patient recovered well and was discharged without any operation-related complications. Biopsy tissues and surgical samples were examined using multiplex immunohistochemistry (mIHC) to reveal the alterations of the tumor microenvironment (TME) after the application of neoadjuvant therapy, especially the inflammatory and immune cells. mIHC of biopsy tissues (**Figures 3E, F**) illustrated intratumoral presence of CD8+ T cells (111/mm2), and high infiltration of FoxP3 (244/mm^2^) and CD68 + CD163 + M2 macrophages (139/mm^2^). In the surgical samples (**Figures 3G, H**), large amounts of CD8+ T cells (511/mm^2^) and tertiary lymphoid structures were observed, while the number of CD68 + CD163 + M2 macrophages (32/mm^2^) and FoxP3+ (60/mm^2^) were substantially decreased. No genetic mutation was detected in the ctDNA of peripheral blood at the fourth postoperative week. The patient started receiving two cycles of adjuvant immuno-chemotherapy (intravenous injection of 200 mg Pembrolizumab, 30 mg/m^2^ lobaplatin + 260 mg/m^2^ nab-paclitaxel, D1, q3w) at the fifth week after surgery. The patient was in good physical condition at the 5-month follow-up visit and refused to accept the one-year single-agent immunotherapy due to financial factors.

## Discussion

Multiple clinical studies (7) proved that neoadjuvant immune monotherapy or combined treatment played a crucial part in the treatment of early-stage NSCLC. The major pathologic response (MPR) rate of neoadjuvant immune monotherapy was 17% - 45% and the pCR was unsatisfactory (0%-16%), while the immuno-chemotherapy resulted in higher MPR (36.9%-83%) and pCR (24%-63%) with manageable AEs and no delay of the surgical schedule. Nevertheless, most of these studies excluded patients with *EGFR+/ALK+*. For advanced NSCLC patients with PD-L1 TPS >50%, immune checkpoint inhibitors(ICIs) prolonged the overall survival(OS) of patients with greater safety, as compared to the platinum-based doublet chemotherapy (8). Under neoadjuvant therapy, patients with high PD-L1 expression also tended to exhibit a higher response rate to immunotherapy. In the Checkmate 816 trial (2), neoadjuvant immuno-chemotherapy was confirmed to be effective in treating stage IB lung cancer, in which the pCR reached 40% and the event-free survival (EFS) risk was lowered by 76% in patients with PD-L1>50%.

*EGFR* or *ALK* gene alterations occur in patients with LUSC, especially non-smoking females, in which TKI treatments present clinical benefits in disease control with a considerably shorter drug-resistant duration in LUSC patients than in lung adenocarcinoma (LUAD) patients (9). A previous study (10) reported that the first-line pembrolizumab treatment was superior to conventional chemotherapy for lung cancer patients with high PD-L1 expression, and the subgroup analysis showed that pembrolizumab was more effective in treating LUSC than non-LUSC. Besides, the subgroup analysis conducted in the Checkmate 816 trial demonstrated the efficacy of preoperative neoadjuvant immono-chemotherapy in both LUAD and LUSC, while the efficacy was more significant in LUAD (HR: 0.5 vs HR: 0.77); this therapy also reduced the risk of recurrence or mortality by 23% (HR = 0.77) among LUSC patients versus chemotherapy alone, exhibiting therapeutic advantage (2). A cell experiment performed by Zhang et al. (11) revealed that higher PD-L1 expression was associated with lower sensitivity to EGFR-TKI of *EGFR*-mutant NSCLC cell lines, which leads to epithelial-to-mesenchymal transition (EMT) induced by the upregulation of Smad3 phosphorylation, potentially contributing to primary drug resistance.

Previous studies have reported that the efficacy of ICIs among NSCLC patients with EGFR mutations was minimal, and even *EGFR* alteration was considered to be related to the hyperprogression induced by ICIs, especially-monotherapy. In a single-center retrospective study (12), the objective response rate (ORR) yielded by immunotherapy of driver mutation-positive lung cancer was only 3.8%; while in another phase 2 clinical trial (NCT02879994), the ORR yielded by first-line pembrolizumab monotherapy was 0% among seven advanced NSCLC patients with *EGFR* mutations and strong PD-L1 expression (13). However, in the trials of IMPOWER 150, KEYNOTE-789, and CheckMate-722 (14–16), the advanced NSCLC patients with high *PD-L1* expression and EGFR mutations responded well to immunotherapy combined with chemotherapy or anti-vascular endothelial growth factor (VEGF)agents. Collectively, ICIs should not be completely excluded in the treatment of *EGFR*-mutated NSCLC.

Several clinical trials also investigated the safety and efficacy of ICIs plus EGFR-TKIs. Jänne et al. (17) revealed that in advanced *EGFR*-mutated NSCLC patients administered with first-line osimertinib plus durvalumab, ORR was 82% (48–98), the median duration of response (DOR) was 7.1 months and median progression-free survival (PFS) was 9.0 months. However, the enrollment in this patient cohort was terminated due to the potential risk of interstitial lung disease (ILD)-related AEs. Considering the increased risk of treatment-related AEs as well as limited efficacy and benefits, the treatment strategy of ICIs plus EGFR-TKIs is possibly unacceptable.

A preclinical study (18) discovered that the activation of the EGFR pathway up-regulated the expression of PD-1, PD-L1, cytotoxic T lymphocyte antigen-4 (CTLA-4), and proinflammatory cytokines, rendering the feature of immunosuppression. This suggested that oncogenic *EGFR* signaling triggered immune escape by remodeling the TME. In the present case, the patient’s postoperative TME showed a significant increase of CD8 + T cells and an evident decrease of FoxP3 and CD68+CD163+ M2 macrophages(**Supplementary Figure**), together with the occurrence of tertiary lymphoid structure. T cells suppressed the anti-tumor functions of immune effector cells and were involved in tumor immune escape (19); M2 macrophages secreted anti-inflammatory cytokines and modulated wound healing to exert the immunosuppressive effects (20), and the tertiary lymphoid structure was associated with a promising prognosis immunotherapy response (21). Immuno-chemotherapy can directly kill cancer cells and regulate immune response to improve the tumor-immune microenvironment (TIME), thereby achieving better efficacy (22).

The ORR of neoadjuvant EGFR- TKI in the EGFR-sensitive mutation population is 48% and early stage NSCLC may reduce the ORR of neoadjuvant EGFR-TKIs (23), and the MPR of neoadjuvant Osimertinib was 15% (5). However the evidence for neoadjuvant targeted therapy especially in these subgroups of EGFR mutation status, PD-L1 expression is still not sufficient. In our case, ICIs were administered prior to EGFR-TKIs, given the low occurrence of driver gene mutation in LUSC, and the limited benefits of EGFR-TKIs. Besides, ICIs were more effective for LUSC, and their combination with chemotherapy might exhibit better efficacy with unknown expression of PD-L1. Due to the imaging remission, the treatment protocol was kept unchanged when the NGS- and IHC suggested high PD-L1 expression and *EGFR* p.L858R mutations accompanied by variation in multiple genes such as *EGFR* amplification. Finally, the patient underwent complete tumor resection and achieved pCR without grade 3-5 AEs during the treatment.

It was reported (24) that after receiving the first-line immuno-monotherapy, an elderly patient with metastatic squamous cell lung cancer harboring both *EGFR* mutation and high PD-L1 expression presented with continuously aggravated conditions and died 6 months later. Additionally, Oguri T et al. (25) reported that atezolizumab monotherapy led to immunotherapy-related hyperprogression in a patient with *EGFR* p.L858R-mutated pulmonary pleomorphic carcinoma. Similar to the above two cases, our case also involved high PD-L1 expression and *EGFR* p.L858R mutations with FOXP3 and M2 macrophages in the preoperative immune microenvironment, which was associated with the development of hyper progressive diseases (HPDs). Yet the tumor was obviously diminished without signs of HPD following the neoadjuvant immuno-chemotherapy, which may be attributed to the effects of immuno-chemotherapy on TIME and the good prognosis the therapy yielded. Still, HPD requires further exploration. Furthermore, Li et al. (26) reported an NSCLC patient with high PD-L1 expression, *EGFR* mutations, and cold tumors who experienced disease progression 3 weeks after neoadjuvant immuno-chemotherapy with LUAD different from LUSC in our case. Huang et al. (27) revealed that the squamous cell carcinoma components of lung adenosquamous adenocarcinoma were likely transformed from adenocarcinoma components and that EGFR-TKIs were effective for advanced *EGFR*-mutant lung adenosquamous carcinoma. Nevertheless, further researches on the immune microenvironment and immunotherapy of lung adenosquamous carcinoma need to be carried out.

Some limitations are present in this case. On the one hand, only one individual case was reported, thus it is tricky to promote this treatment protocol. On the other hand, due to the heterogeneity of tumor tissues, initial findings in pathologic biopsy were potentially inconsistent with the final ones, which means that adenosquamous carcinoma or inconsistent gene expression were probable, posing a potential risk for the selection of optimal neoadjuvant therapy modality.

Taken together, we reported a patient with stage IB NSCLC and LUSC harboring high PD-L1 expression and *EGFR* mutations who achieved pCR after two courses of neoadjuvant therapy of platinum-based chemotherapy plus pembrolizumab. This case illustrated the feasibility and effectiveness of neoadjuvant immuno-chemotherapy in treating early LUSC with *EGFR* mutations and high PD-L1 expression. Nonetheless, the conclusion requires validation in more patient samples. Moreover, therapeutic genes and biomarkers need to be further explored to select the optimal treatment protocol for clinical scenarios with co-existing PD-L1 upregulation and *EGFR* sensitivity mutation.

## Data availability statement

The datasets presented in this article are not readily available because of ethical/privacy restrictions. Requests to access the datasets should be directed to the corresponding author.

## Ethics statement

The studies involving human participants were reviewed and approved by Chongqing General Hospital of ethics committee. The patients/participants provided their written informed consent to participate in this study.

## Author contributions

ZM was responsible for the organization and coordination of the case. XX was the attending physician. XX, ZS, and DH contributed to data collection. XX and DF were responsible for original draft preparation. All authors contributed to the article and approved the submitted version.

## Conflict of interest

DF and DH were employed by the 3D Medicines Inc.

The remaining authors declare that the research was conducted in the absence of any commercial or financial relationships that could be construed as a potential conflict of interest.

## Publisher’s note

All claims expressed in this article are solely those of the authors and do not necessarily represent those of their affiliated organizations, or those of the publisher, the editors and the reviewers. Any product that may be evaluated in this article, or claim that may be made by its manufacturer, is not guaranteed or endorsed by the publisher.
