# Supplementary figures and images for: EGFR mutations and high PD-L1 expression of lung squamous cell carcinoma patients achieving pCR following neoadjuvant immuno-chemotherapy: Case report
Source: Front Oncol. 2022 Oct 19;12:1008932. doi: 10.3389/fonc.2022.1008932 (PMC9627657; doi:10.3389/fonc.2022.1008932)

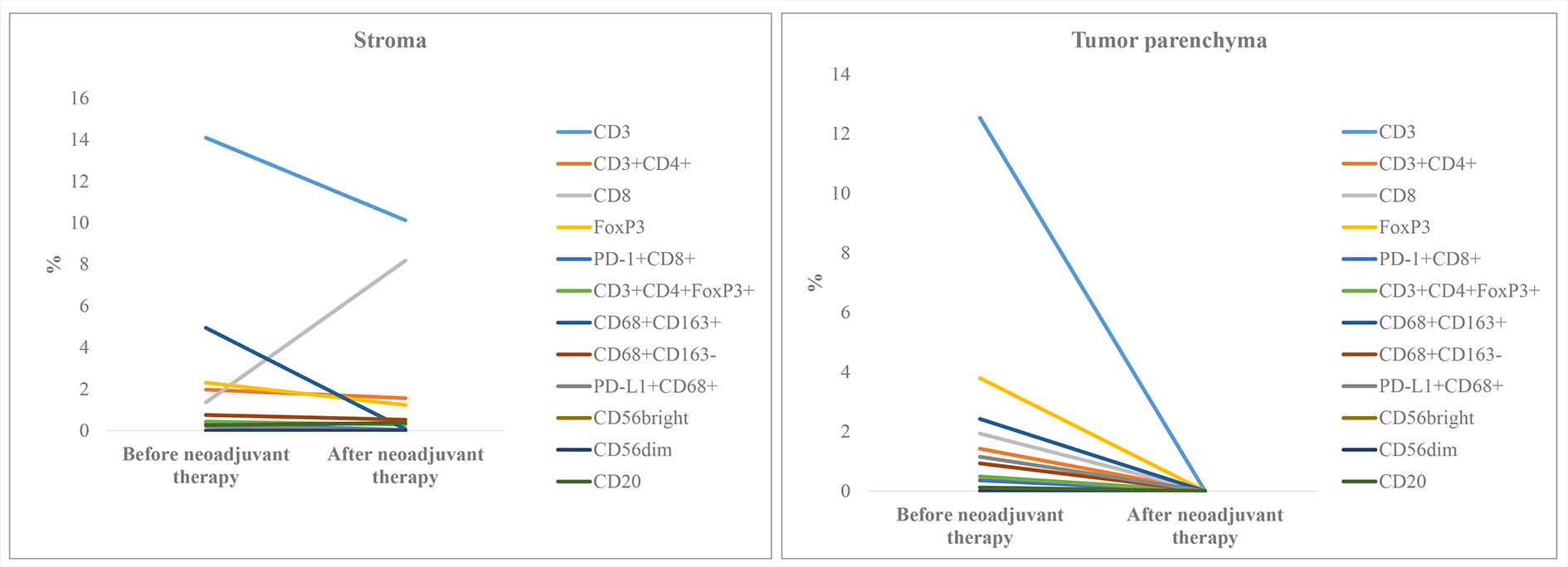

Supplement: Supplementary file 1 [file Image_1.tif]
